# Case‐malformed signal detection and prioritisation using EUROmediCAT data for pharmacovigilance in pregnancy

**DOI:** 10.1002/bcp.70645

**Published:** 2026-07-01

**Authors:** Hannah Johnson, Helen Dolk, Maria Loane, Christine Damase‐Michel, Joanne Given, Hedvig Nordeng, Jorieke E. H. Bergman, Iain Carey, Elly Den Hond, Ester Garne, Florence Rouget, Lea Bruneau, Isabelle Monier, Anke Rissmann, Mary O'Mahony, Miriam Gatt, Renée Lutke, Joanna Sichitiu, Elisa Ballardini, Alessio Coi, Clara Cavero Carbonell, David Tucker, Joan Morris

**Affiliations:** ^1^ City St George's (School of Health & Medical Sciences) London UK; ^2^ School of Medicine Ulster University Londonderry UK; ^3^ Institute of Nursing and Health Research Ulster University Northern Ireland UK; ^4^ Faculté de Médecine CERPOP UMR INSERM, Centre Hospitalier Universitaire Université Toulouse Toulouse France; ^5^ Pharmacoepidemiology and Drug Safety Research Group, Department of Pharmacy, Faculty of Mathematics and Natural Sciences University of Oslo Oslo Norway; ^6^ Department of Child Health and Development Norwegian Institute of Public Health Oslo Norway; ^7^ EUROCAT Northern Netherlands, Department of Genetics University of Groningen, University Medical Centre Groningen Groningen the Netherlands; ^8^ Provincial Institute of Hygiene (PIH) Antwerp Belgium; ^9^ Department of Paediatrics and Adolescent Medicine, Lillebaelt Hospital University of Southern Denmark Kolding Denmark; ^10^ Brittany Registry of Congenital Anomalies, CHU Rennes, Univ Rennes, Inserm, EHESP, Irset (Institut de recherche en santé, environnement et travail) Rennes France; ^11^ The Reunion Registry of Congenital Malformations (REMACOR), Department of Public Health and Research Support University Hospital of La Réunion Saint Pierre Reunion Island France; ^12^ National Institute of Health and Medical Research INSERM, Clinical Epidemiology, Department of Public Health and Research Support University Hospital of La Réunion Saint Denis Reunion Island France; ^13^ Centre for Research in Epidemiology and Statistics, Obstetrical, Perinatal and Pediatric Life Course Epidemiology Research Team, Inserm, INRAE, Université Paris Cité and Université Sorbonne Paris Nord Paris France; ^14^ Malformation Monitoring Centre Saxony‐Anhalt, Medical Faculty Otto‐von‐Guericke‐University Magdeburg Magdeburg Germany; ^15^ Department of Public Health St Finbarr's Hospital Cork Ireland; ^16^ Malta Congenital Anomalies Registry Directorate for Health Information and Research Pietà Malta; ^17^ Ultrasound and Fetal Medicine Unit, Department of Women‐Mother‐Child Lausanne University Hospital and University of Lausanne Lausanne Switzerland; ^18^ Neonatal Intensive Care Unit, University Hospital of Ferrara, IMER Registry (Emilia Romagna Registry of Birth Defects), Department of Medical Sciences University of Ferrara Ferrara Italy; ^19^ Unit of Epidemiology of Rare Diseases and Congenital Anomalies, Institute of Clinical Physiology National Research Council Pisa Italy; ^20^ Rare Disease Research Unit Foundation for Promotion of Health and Biomedical Research of Valencian Region (FISABIO) Valencia Spain; ^21^ Research, Data & Digital Directorate Public Health Wales Swansea UK

**Keywords:** congenital anomaly, disproportionality analysis, EUROmediCAT, pregnancy, signal detection

## Abstract

**Aim:**

Many women take medications during pregnancy. However, the risk to the fetus from most medications is uncertain. Congenital anomalies are one of the leading causes of infant death and contribute to long‐term disability. Signal detection methods can be used to systematically identify possible medication–anomaly associations that require further investigation.

**Methods:**

Data on first trimester medication exposures in pregnancies with a congenital anomaly reported to 14 EUROmediCAT registries with a birth year of 2005–2018 were analysed. Case‐malformed disproportionality analysis identified medication–anomaly signals using a disproportionality signal detection method. Identified signals were then compared to previous EUROmediCAT signal detection studies and ranked based on a proxy for the severity of impact at a population level using the number of estimated excess congenital anomaly cases within the exposed population and the average additional time in hospital over the first year of life. Generalized linear mixed models were used to assess confounding by birth year and registry within the top 20 ranked signals.

**Results:**

1611 medication–outcome pairs with at least three observations were analysed, and 153 signals for 63 different medications were identified. Of the top 20 ranked signals, 10 (involving nine medications) were prioritized for further independent investigation.

**Conclusion:**

The signals identified here are hypothesis forming only. Independent studies that adequately account for confounding are subsequently needed to evaluate if the identified signals are causal. Further investigation of signals with low population impact, but high potential individual risk is also recommended.

**What is already known about this subject**

- Many women take medications during pregnancy; however, the fetal teratogenic risk for most medications is uncertain.
- Congenital anomalies affect around 2%–3% of births and are one of the leading causes of infant death in Europe.
- Signal detection methods provide a systematic approach to identification of medications that require further investigation.

**What this study adds**

- 153 medication–anomaly associations were identified as signals and ranked based on severity of impact at a population level.
- Of the top 20 ranked signals, 10 signals (involving nine medications) were recommended for further investigation.
- Identified signals are not proof of harm. Further studies that consider confounding are required to assess causality.

## 1 INTRODUCTION

An estimated 65%–95% of women take prescribed or over‐the‐counter medications during their pregnancy across Europe and the United States.^1^, ^2^, ^3^, ^4^ Medication use during pregnancy has been increasing over time.^5^ However, the risk to the fetus for most medications is uncertain, in part due to exclusion of pregnant women from clinical trials.

Observational studies are often used to evaluate safety of medications after concerns over a specific medication are raised. However, observational data can also be used to systematically identify possible concerns that should be evaluated. Quantitative signal detection methods scan through all medication–outcome pairs within a dataset to find ‘signals’: pairs where the outcome occurs significantly more frequently in those exposed to the specific medication than those not exposed. Signals do not indicate the medication causes the increased risk of the outcome, but the relationship should be explored with further research using independent data.

For some medications, first trimester in‐utero exposure increases the risk of congenital anomalies in the fetus. Congenital anomalies are structural or functional malformations that arise during development in utero and are estimated to effect around 2%–3% of births in Europe (including stillbirths and terminations).^6^ Congenital anomalies are now one of the leading causes of infant death^7^ and contribute to long‐term disability.

EUROCAT is a network of European population‐based congenital anomaly registries that aims to facilitate the early warning of teratogenic exposures.^8^ EUROmediCAT consists of EUROCAT registries that provide data on first trimester medication exposure^9^ using World Health Organization Anatomical‐Therapeutic‐Chemical (WHO‐ATC) coding. EUROmediCAT first applied signal detection methods in 2016 using data from birth years 1995 to 2011.^10^, ^11^ Since then, additional data have been collected, and further improvements made to the signal detection methods.

This study aimed to identify medication safety signals in pregnancy in EUROmediCAT using a novel signal detection method.^12^ These signals were compared to signals identified from previous EUROmediCAT signal detection studies^10^, ^11^, ^13^ and ranked based on severity of anomaly and number of excess congenital anomaly cases occurring in the population of pregnant women exposed to the specific medication. The estimated associations of highly ranked signals were adjusted for birth year and registry and evaluated considering what is already known within the literature.

## 2 METHODS

### 2.1 Signal detection

First, we performed case‐malformed signal detection analysis using the novel Information Component‐Bicluster‐Bayesian Hierarchical Model (IC‐Bic‐BHM) method^12^ within the EUROmediCAT population‐based registry data.

EUROmediCAT registries record major congenital anomalies diagnosed in all livebirths, fetal deaths from 20‐week gestation, and terminations following prenatal diagnosis.^14^ The study population included all diagnoses recorded within 14 EUROmediCAT registries with a birth year between 2005 and 2018. The study was approved by St George's, University of London Research Ethics Committee (approval number 2022.0074). Information on informed consent procedures for individual registries can be found on the Member Registries section of the EUROCAT website.^15^ Although data were available prior to 2005, coding of drug exposures using ATC code is only available from 2005 onwards. Livebirths, fetal deaths and terminations were excluded from this study if recorded with a genetic or chromosomal anomaly according to EUROCAT guide 1.4^14^ or were diagnosed with an isolated hip dislocation, as these are generally considered not to arise from teratogenic medication exposures. Terminations from Emilia Romagna, and terminations and fetal deaths from Valencia region, were excluded as medication exposures are not recorded for these outcomes within these registries. Malta and Cork and Kerry registries do not record terminations.

A case can be exposed to multiple medications in utero. Included exposures were fifth‐level WHO‐ATC codes, with at least three observations within the dataset. Fourth‐level codes were included where no fifth‐level code is defined within the WHO‐ATC hierarchy (i.e. D02AX other emollients and protectives; G01BA antibiotics and corticosteroids; and H03CA iodine therapy). Vitamins, minerals and nutrients were not analysed (Table S1). WHO‐ATC codes are continually updated^16^; older codes were updated to newer codes where possible. WHO‐ATC codes are classified by body system, so a single medication can have multiple codes (e.g. acetylsalicylic acid has multiple codes dependant on the target body system). Each WHO‐ATC code was considered a separate medicinal product, rather than combining based on chemical substance. For each specific medication, exposed were those recorded as taking the specific medication in the first trimester of pregnancy (defined by EUROmediCAT as the first day of the last menstrual period up to the 12th week of gestation), and unexposed were those not recorded as taking the specific medication in the first trimester of pregnancy (note that this includes individuals with no medications recorded).

Most included registries primarily use medical records as the source of exposure information^17^ (rather than prescription or dispensation data) and therefore may have under‐reporting of some types of medication, particularly those used to treat acute illness.^18^ Previous EUROmediCAT signal detection studies excluded cases unexposed to any medications of interest.^10^, ^13^ In this study, cases were not excluded if unexposed to any medication of interest to allow for increased power to detect signals.

Congenital anomaly diagnosis is recorded using the International Classification of Disease 10th revision with British Paediatric Association extension codes (ICD‐10‐BPA)^19^ and mapped to EUROCAT anomaly subgroups.^14^ A case can have multiple anomalies recorded. Included outcomes were anomaly subgroups with at least three observations within the dataset. Known teratogenic syndromes, chromosomal anomalies and genetic syndromes, hip dislocation, organ system groups (e.g. nervous system anomalies; respiratory anomalies; gastrointestinal anomalies) and composite groups (e.g. vascular disruption anomalies; laterality anomalies) were not analysed (Table S2). Ascertainment of cases is expected to be high.^20^ For the sex‐specific diagnoses of posterior urethral valves and hypospadias, the dataset was restricted to cases registered as male.

Signals were identified using the IC‐Bic‐BHM signal detection method, which identifies groups of medications and groups of anomalies from within the data and uses information sharing across these groups to inform results (see reference for full details of method application to this data).^12^ An IC‐Bic‐BHM statistic greater than 1 indicates the medication–anomaly is observed more frequently than expected. For example, a statistic of 2 indicates the medication–anomaly is observed two times as frequency as expected. A value of 1 therefore indicates no increase in frequency. All signals were defined as medication–anomaly pairs where the lower 95% credible interval exceeds 1. The Bayesian methodology applies both shrinkage to the estimates, in addition to being able to analyse low counts for rare anomalies and medications.

### 2.2 Comparison of previous signal detection studies in EUROmediCAT

Medication–outcome signals reported within the 1995–2011 signal detection paper (including those not meeting false discovery rate criteria)^10^ and the 1995–2015 signal detection paper^13^ were extracted and compared with signals identified by the IC‐Bic‐BHM. Signals from the previous studies were not analysed by the IC‐Bic‐BHM if they did not meet the above inclusion criteria.

### 2.3 Signal prioritization and ranking

One criterion for assessing potential impact of further investigations is population‐level effect. This depends upon the severity of the anomaly and the number of potential congenital anomalies that could be prevented, assuming the cause of the increased risk (either medication or confounding factor) can be identified and then modified or prevented in pregnancy. Identified signals were prioritized based upon the severity of anomaly according to average number of inpatient hospital days in the first year of life. The number of potential congenital anomalies that could be prevented was estimated from the number of excess congenital anomaly cases occurring in the population of pregnant women exposed to the specific medication.

The average number of days in hospital over the first year of life by EUROCAT anomaly group was taken from Morris et al.^21^ Estimates for anomaly groups not reported within the paper were obtained through author correspondence (personal communication from Professor Joan Morris, City St George's, University of London). Estimates were not available for anencephalous and similar, bilateral renal agenesis and congenital constriction bands. As anencephaly and bilateral renal agenesis are lethal anomalies, 365 days was used to reflect severity. For congenital constriction bands, the average number of days for all anomalies including chromosomal and genetic was used (17.4 days).

An estimated number of exposures for each medication per 100 000 pregnancies was calculated using the proportion of medications recorded within included cases. The number of exposed pregnancies required to result in one additional anomaly^22^ was estimated for each medication–anomaly pair identified as a signal. The estimated number of excess congenital anomaly cases per 100 000 pregnancies was then calculated as the estimated number of exposures per 100 000 pregnancies divided by the number needed to harm (i.e. the estimated number of pregnant women who would need to take the medication to result in one additional anomaly).

To estimate the total additional hospital time, the number of excess congenital anomaly cases per 100 000 pregnancies was multiplied by the average number of days in hospital during the first year of life. This reflects the added hospital burden among infants of pregnant women exposed to the medication, attributed to the specified anomaly. Signals were then ranked, with a lower rank corresponding to a larger estimated total additional hospital time.

Of identified signals, the top 20 were further analysed to check for confounding by birth year and registry. The R package lme4^23^, ^24^ was used for generalized linear mixed models with outcome: specific anomaly (yes/no); covariates: specific medication (yes/no); centred calendar birth year (indices); registry (categorical); with random effects for the intercept and slope of registry; and logit links. For each investigated signal, registries without any recorded exposures to the medication of interest were excluded (Table S3).

The top 20 signals were annotated with information on concurrent exposures and outcomes with a frequency of at least 3. Cases were considered to have a concurrent exposure if recorded with any additional included WHO‐ATC code (Table S1). Cases were considered to have a concurrent outcome if recorded as belonging to any additional EUROCAT anomaly subgroups (Table 1) The number of cases without any additional EUROCAT anomaly subgroups (isolated cases) is also reported.

Signals were not recommended for further investigation if the adjusted odds ratio was not statistically significant; the medication–anomaly pair is already a well‐described teratogenic effect; or the medication–anomaly pair is already a well‐described indication effect attributable to the underlying maternal indication (i.e. diabetes, opioid dependence).

For signals recommended for further investigation, literature was reviewed using REPROTOX and PubMed searches. REPROTOX is a database containing referenced summaries of effects of medications, chemicals, biologics and physical agents on pregnancy, reproduction, lactation and development.^25^ Active substances were searched within REPROTOX to identify relevant original research.

## 3 RESULTS

### 3.1 Signal detection

A total of 113 446 cases were recorded (Table 1). Of these, 25 933 were excluded (21 241 chromosomal or genetic cases; 2239 isolated hip dislocation; and 2453 terminations or stillbirths from registries unable to provide medication data for these outcomes), leaving 87 513 for analysis.

**TABLE 1:** Registry data included in EUROmediCAT analysis.

Registry	Birth years included	Total number of cases for included birth years	Number included in analysis (%)
Total		113 446	87 513 (77%)
Antwerp, Belgium	2005–2017	7050	5628 (80%)
Emilia Romagna, Italy	2005–2018	13 183	9544 (72%)
Northern Netherlands, Netherlands	2005–2018	7312	5806 (79%)
Funen, Denmark	2005–2018	2049	1552 (76%)
Tuscany, Italy	2005–2018	8588	6670 (78%)
Vaud, Switzerland	2005–2018	4107	3055 (74%)
Cork and Kerry, Ireland	2005–2018	3837	2901 (76%)
Malta, Malta	2005–2018	1798	1559 (87%)
Wales, United Kingdom	2005–2018	16 445	13 143 (80%)
Saxony Anhalt, Germany	2005–2018	7544	6586 (87%)
Paris, France	2005–2017	11 022	7994 (73%)
Isle de la Reunion, France	2005–2018	6251	5031 (80%)
Valencian Region, Spain	2007–2018	13 927	9528 (68%)
Brittany, France	2011–2018	10 333	8516 (82%)

*Note*: See methods for reasons for exclusions.

A total of 536 medications and 62 anomalies were analysed. The most frequently reported medication exposures were levothyroxine (2270, 2.6% of pregnancies); paracetamol (1476, 1.7% of pregnancies) and progesterone (798, 0.9% of pregnancies). The most frequently recorded congenital anomalies were ventricular septal defect (15 517, 18%), hypospadias (7773, 8.9%) and atrial septal defect (6060, 6.9%).

A total 1611 medication–outcome pairs with at least three observations were analysed. Pairs with the largest observed frequency included ventricular septal defect with levothyroxine sodium (*N* = 452), ventricular septal defect with paracetamol (*N* = 239) and hypospadias with levothyroxine sodium (*N* = 197). In total, 153 signals for 63 different medications and 45 different anomalies were identified (Table S4).

### 3.2 Comparison of signal detection in EUROmediCAT

A total of 45 medication–anomaly signals were reported within the 1995–2011 study; and 49 medication–anomaly signals were reported within the 1995–2015 study. Most signals from the 1995–2011 and 1995–2015 analyses were not analysed by the IC‐Bic‐BHM (Figure S1) due to differences in inclusion–exclusion criteria, the number of included cases, signal detection method, exposures and outcomes (Table S5). Only three of the 43 signals in the 1995–2011 study were included in this analysis; all three were also identified by the IC‐Bic‐BHM (Figure S1). Similarly, only 14 of the 49 signals in the 1995–2015 analysis were included in this analysis, of which eight were identified by the IC‐Bic‐BHM. For the six signals not identified by the IC‐Bic‐BHM, point estimates were similar (Table S6). In total, 143 new signals were identified by the IC‐Bic‐BHM that were not found in previous analysis.

### 3.3 Signal prioritization and ranking

Of medications with an identified signal using the new IC‐Bic‐BHM method, proportion of pregnancies recorded with a first trimester exposure ranged from seven per 100 000 for the antipsychotic medication flupentixol to 2540 per 100 000 for levothyroxine sodium. For identified signals, the estimated number needed to harm ranged from 1.5 to 299, while the estimated number of excess congenital anomaly cases per 100 000 pregnancies ranged from 2.4 to 68 (Table 2).

**TABLE 2:** Ranking of top 20 signals.

Medication–anomaly pair		Signal			Ranking					
ATC code/substance name	AL code/anomaly name	Observed Frequency	Expected Frequency	IC‐Bic‐BHM (95% credible interval)	Estimated number of exposures per 100 000 pregnancies	Number needed to harm	Estimated additional cases per 100 000 pregnancies	Average hospital time in first year of life for anomaly (days)	Estimated additional hospital time per 100 000 pregnancies (days)	Rank
A02BB01/misoprostol	AL4/anencephaly	5	0.3	15 (5.2–32)	27	5.1	5.3	365	1959	1
C09CA03/valsartan	AL53/bilateral renal agenesis	4	0.1	22 (3.1–79)	17	3.8	4.5	365	1642	2
R01AD01/beclometasone	AL53/bilateral renal agenesis	3	0.2	1.2 (1.7–22)	54	17	3.2	365	1168	3
H03AA01/levothyroxine sodium	AL41/oesophageal atresia with or without fistula	39	23	1.7 (1.2–2.3)	2540	136	19	56	1047	4
H03AA01/levothyroxine sodium	AL24/tetralogy of Fallot	48	28	1.7 (1.3–2.3)	2540	106	24	35	829	5
H03AA01/levothyroxine sodium	AL21/ventricular septal defect	452	394	1.1 (1.0–1.3)	2540	38	68	11	739	6
N02BE01/paracetamol	AL50/gastroschisis	22	13	1.7 (1.1–2.5)	1683	156	11	65	700	7
R03AC02/salbutamol	AL50/gastroschisis	13	5.2	2.4 (1.3–4.0)	695	78	8.9	65	578	8
C08CA05/nifedipine	AL41/oesophageal atresia with or without fistula	10	2.1	4.5 (2.3–8.0)	233	26	9.0	56	507	9
G03DB01/dydrogesterone	AL22/atrial septal defect	36	13	2.8 (2.0–3.9)	207	7.7	27	18	494	10
G03DA04/progesterone	AL22/atrial septal defect	74	54	1.4 (1.1–1.7)	890	39	23	18	425	11
H02AB01/betamethasone	AL22/atrial septal defect	34	14	1.8 (1.3–2.6)	231	10	23	18	422	12
N03AG01/valproic acid	AL6/spina bifida	14	2.3	5.8 (3.3–9.5)	154	12	13	26	349	13
A10AB05/insulin aspart	AL19/transposition of great vessels	10	2.7	3.5 (1.7–6.2)	233	28	8.3	42	346	14
N03AX09/lamotrigine	AL43/atresia or stenosis of other parts of small intestine	4	0.7	5.1 (1.5–12)	170	45	3.8	91	344	15
J01CA01/ampicillin	AL41/oesophageal atresia with or without fistula	7	1.8	3.7 (1.6–7.2)	198	33	6.0	56	335	16
B01AC06/acetylsalicylic acid	AL22/atrial septal defect	40	25	1.6 (1.1–2.1)	411	24	17	18	318	17
N03AG01/valproic acid	AL22/atrial septal defect	24	9.3	2.5 (1.7–3.7)	154	9.2	17	18	309	18
H03CA/iodine therapy	AL8/severe microcephaly	24	7.3	3.2 (2.1–4.7)	623	32	19	15	296	19
H03CA/iodine therapy	AL55/congenital hydronephrosis	56	32	1.7 (1.3–2.2)	623	23	28	11	290	20

*Note*: Estimated additional cases, average time in hospital in 1st year of life and estimated additional hospital time per 100 000 pregnancies rounded after calculation. Lethal anomalies (anencephaly and bilateral renal agenesis) given 365 days in hospital. For congenital constriction bands, the average number of days for all anomalies including chromosomal and genetic was used (17.4 days).

Abbreviation: IC‐Bic‐BHM, Information Component‐Bicluster‐Bayesian Hierarchical Modelling.

All top 20 signals had some cases with concurrent exposures, although none could be explained by concurrent exposure to known teratogenic medications (Table S7).

Of the top 20 signals, based on population impact, 10 signals were recommended for further investigation. After assessing the top 20 signals with generalized linear models to account for confounding by registry and birth year, five did not have a statistically significant increased odds ratio; four were for well described teratogenic effects; and one was for a well described indication effect (i.e. diabetes) (Table S3). The top ranked signal recommended for further investigation was for bilateral renal agenesis with nasal beclometasone (Table 3).

**TABLE 3:** Recommendation for top 20 signals.

Medication–anomaly pair	
Substance name (ATC code)	Anomaly name (AL code)
**Recommended for further investigation**	
1 Beclometasone (R01AD01)	Bilateral renal agenesis (AL53)
2 Levothyroxine (H03AA01)	Oesophageal atresia with or without fistula (AL41)
3 Levothyroxine (H03AA01)	Tetralogy of Fallot (AL24)
4 Paracetamol (N02BE01)	Gastroschisis (AL50)
5 Salbutamol (R03AC02)	Gastroschisis (AL50)
6 Nifedipine (C08CA05)	Oesophageal atresia with or without fistula (AL41)
7 Betamethasone (H02AB01)	Atrial septal defect (AL22)
8 Lamotrigine (N03AX09)	Atresia or stenosis of other parts of small intestine (AL43)
9 Ampicillin (J01CA01)	Oesophageal atresia with or without fistula (AL41)
10 Iodine therapy (H03CA)	Severe microcephaly (AL8)
**Not recommended for further investigation: Odds ratio not statistically significant once adjusted for birth year and registry**	
Levothyroxine sodium (H03AA01)	Ventricular septal defect (AL21)
Dydrogesterone (G03DB01)	Atrial septal defect (AL22)
Progesterone (G03DA04)	Atrial septal defect (AL22)
Acetylsalicylic acid (B01AC06)	Atrial septal defect (AL22)
Iodine therapy (H03CA)	Congenital hydronephrosis (AL55)
**Not recommended for further investigation: Known teratogenic effect**	
Misoprostol (A02BB01)	Anencephaly (AL4)
Valsartan (C09CA03)	Bilateral renal agenesis (AL53)
Valproic acid (N03AG01)	Spina bifida (AL6)
Valproic acid (N03AG01)	Atrial septal defect (AL22)
Insulin aspart (A10AB05)	Transposition of great vessels (AL19)

*Note*: Numbers for recommended signals indicate their rank after exclusions.

## 4 DISCUSSION

Application of the new IC‐Bic‐BHM method identified 153 medication–anomaly signals for 63 medications and 45 anomalies. These signals were prioritized based upon estimated impact at a population level. The top 20 were further annotated, and 10 (involving nine medications) were recommended for further investigation.

### 4.1 Signal detection

Identified signals only indicate a statistically significant increase in medication exposure within the population of fetuses with the specific anomaly, compared to the population of fetuses without the specific anomaly. Signals may be due to confounding factors that were not controlled for during this analysis, including the indication for the medication (confounding by indication). However, the identification of a teratogenic co‐exposure through the investigation of a signal may be just as important as identification of a teratogenic medication.

### 4.2 Comparison of signal detection in EUROmediCAT

There was little overlap between the medication–anomaly pairs analysed by the IC‐Bic‐BHM and previous EUROmediCAT signal detection studies. In this study, analysis was only performed for fifth‐level WHO‐ATC medications. Previous study results were primarily for fourth‐level WHO‐ATC medications as frequencies were too low to investigate most fifth‐level medications. Differences in frequencies may also be explained by the inclusion/exclusion of different birth years. Where there was overlap, signals were mostly consistent across the three analyses. The IC‐Bic‐BHM identified a far larger number of signals than previous studies, which may be ascribed to both to the larger number of included cases resulting in greater power, and less strict adjustment for multiple comparisons through Bayesian shrinkage compared to the use of a false discovery rate.

### 4.3 Signal prioritization

Signals were prioritized based upon a proxy for severity at a population level. The ranking assumed prevalence of medication exposures within the EUROmediCAT dataset are representative of the wider population of pregnant women across Europe, which may not be true. Exposures to teratogenic medications may be over‐represented among fetuses with an anomaly and therefore over‐represented within the EUROmediCAT dataset. As such, this approach may have inflated estimates of exposure frequency. A minority of anomalies did not have estimates for additional time spent in hospital in the first year of life. Several highly ranked signals were for digestive tract atresias and abdominal wall defects, which confer far greater time in hospital than other anomalies. The three most highly ranked signals were for lethal anomalies, which were assigned a default of 365 days.

Associations between medication use during pregnancy and congenital anomalies are rarely considered from a population health viewpoint, instead focusing on information that is useful for counselling an individual during pregnancy. Small increased risks are more likely to be explained by confounding factors. However, genuine small increased risks may have an important impact at the population level if the medication is commonly prescribed. Some of the top ranked signals were for relatively low increased risks with the use of levothyroxine sodium. However, this medication is commonly prescribed in pregnancy (2.6% of pregnancies in this dataset) and therefore had high estimated excess cases. Several well‐known teratogens, valproic acid, misoprostol, valsartan and diabetes (for which we analysed the proxy insulin) are included among the top 20 signals in terms of population impact, even though they do not require further investigation as to causality.

This method of prioritization will miss large risks for rarely used medications. Women who take rare medications have an equal right to prioritization on an individual risk basis. Signals that do not have a high population impact, but where there are large relative risks relevant to the individual exposure, should also be prioritized for further investigation but are not the subject of this paper. For example, 23 of the 153 signals had a relative risk of at least 3 and observed frequency of at least 6, of which only five were within the top 20 signals based on population impact.

### 4.4 Evaluation of signals with available evidence

Review of the literature for medications recommended for further investigation highlighted the lack of studies that adequately control for the indication of the medication, studies with large enough sample sizes for investigation into specific anomalies, studies that contain prospectively collected medication data, and studies that do not collate active ingredients together when ascertaining overall or specific risks.

#### 4.4.1 Nasal beclometasone and bilateral renal agenesis

This signal is based on an observed frequency of 3 and may be due to chance or confounding factors. Limited amounts of nasal beclometasone reach systemic circulation, and therefore, fetal exposure is unlikely. For other routes of administration, no previous studies have reported an increased risk of any anomaly with the use of beclometasone. Most studies have investigated the combined effect of corticosteroids or the combined effect of inhaled asthma medications rather than individual active ingredients. One prospective randomized controlled trial compared beclometasone to theophylline and found no difference in major anomalies, although the primary outcome was asthma exacerbations and frequencies were too low to compare specific anomalies.^26^ A 2014 systematic review of beclometasone use in pregnancy found articles were too heterogeneous to perform a meta‐analysis.^27^ Further studies with larger sample sizes are needed to investigate the risk of specific anomalies for systemic exposure to beclometasone.

#### 4.4.2 Levothyroxine sodium and oesophageal atresia and tetralogy of Fallot

Two of the top ranked signals were for levothyroxine sodium. One observational study reported associations between maternal thyroid disease in pregnancy and specific types of congenital anomaly.^28^ Two studies have reported small increased risks of overall anomalies with the use of thyroid hormones (odds ratio 1.13), as well as increased risks for various specific anomalies (odds ratios for anencephaly 1.68; holoprosencephaly 2.48; hydrocephaly 1.77; and atresia of the small intestine 1.81).^29^, ^30^ However, these studies either did not report on oesophageal atresia or tetralogy of Fallot; or the frequency of observations was far smaller than in this analysis. These signals may be due to confounding by indication, and further research is required to fully understand the relationship between thyroid hormone levels and congenital anomalies while keeping in mind the importance of adequate treatment for hypothyroidism in pregnancy for both mother and child.^31^

#### 4.4.3 Paracetamol and gastroschisis and anorectal atresia and stenosis

Several observational studies found no overall increased risk of congenital anomalies,^32^, ^33^, ^34^ or no risk for specific congenital anomalies^33^, ^34^, ^35^, ^36^ with the use of paracetamol during pregnancy. One previous EUROmediCAT case‐malformed control study found a weak association between paracetamol and gastroschisis.^37^ Paracetamol may be obtained by prescription, or over the counter, and is likely to be under‐reported in EUROmediCAT data. As our analyses is case‐malformed, there is unlikely to be a reporting bias even for the two of 14 registries within these analyses that collect medication exposures retrospectively. Prescriptions of paracetamol may be confounded by multi‐morbidity and low socio‐economic status. As such, paracetamol cannot be adequately investigated in this setting. However, as most studies fail to adequately control for the indications for paracetamol (illness and disease that cause fever, inflammation and/or pain) or use retrospective self‐reported data, we felt it important to recommend further research in this area.

#### 4.4.4 Salbutamol and gastroschisis

Most observational studies have investigated the combined effect of inhaled asthma medications, rather than the individual active ingredients.^38^, ^39^ Of studies that investigated salbutamol individually, one showed a small overall increased risk of congenital anomalies,^40^ and two showed increased risks for specific congenital anomalies (i.e. any cardiac defect and orofacial clefts).^40^, ^41^ However, the anomalies identified by these two studies were different. Although not independent to this study, two previous studies used EUROmediCAT data to assess the risk of gastroschisis with salbutamol exposure including verification of timing of exposure: One found increased odds of exposure to salbutamol in those with gastroschisis,^42^ while the other found no increased odds.^37^ As untreated asthma is itself associated with congenital anomalies,^43^ this signal may be due to confounding by indication. Further research is required to understand any relationship between asthma and congenital anomaly risk and asthma medications and congenital anomaly risk.

#### 4.4.5 Nifedipine and oesophageal atresia with or without fistula

Some observational studies have assessed the combined effect of calcium channel blockers, rather than individual active ingredients.^44^ Two studies found associations with specific anomalies,^45^, ^46^ one of which also reported 3.8 times the odds of in‐utero exposure to nifedipine in those with oesophageal atresia. These studies had small numbers of observed anomalies and did not distinguish between maternal hypertension and the use of nifedipine.

#### 4.4.6 Betamethasone and atrial septal defect

Many observational studies have investigated the combined effect of glucocorticoids or corticosteroids, rather than individual active ingredients of which only two explicitly report the inclusion of exposures to betamethasone.^47^, ^48^ Betamethasone can be used in cases of premature delivery. Premature births are themselves associated with secundum atrial septal defect. EUROmediCAT registries are advised to only record first trimester exposures; however, we did not verify timing of medication in individual exposed cases. Further studies are needed to assess the individual risks and comparative risks of different types of glucocorticoids and corticosteroids.

#### 4.4.7 Lamotrigine and atresia or stenosis of other parts of small intestine

A comprehensive systematic review found a slight increased risk of major congenital anomaly in women who took lamotrigine in pregnancy, compared to women who did not have epilepsy, but no increased risk when compared to women with epilepsy.^49^ Other than nervous system anomalies, no specific anomalies were identified within this systematic review as having increased risks. Lamotrigine has been extensively compared to other anti‐epileptics and has been found to be associated with a lower risk of major congenital anomalies than other anti‐epileptic medications.

#### 4.4.8 Ampicillin and oesophageal atresia with or without fistula

Some studies found increased risks of specific anomalies with the use of ampicillin (i.e. congenital heart defects and cleft palate),^50^, ^51^ but the anomalies identified by these two studies, and the current signals, are different. Results may be confounded by the underlying maternal infection. Compared to other medications, there has been little research into the risks of specific congenital anomalies with the use of ampicillin.

#### 4.4.9 Iodine therapy and microcephaly

No controlled observational studies were found investigating iodine therapy (e.g. Lugol's solution) and the risk of congenital malformations, although there are several studies detailing the effects of iodine deficiency and excess. As with levothyroxine, further research is required to fully understand the relationship between iodine levels and congenital anomalies while keeping in mind the importance of adequate treatment for hyperthyroidism in pregnancy.

### 4.5 Strengths and limitations

A strength of this study was the use of a large international standardized database of medication use in pregnancy and congenital anomalies, providing power to detect associations between these medications and rare outcomes.

However, there are several limitations. We did not consider mortality in the ranking of anomaly severity, and prioritization was based only on population impact, rather than individual risk. Further work is needed to assess lower ranked signals in turn. We also did not consider confounding or biological plausibility as part of the ranking system. Only the top 20 ranked signals were assessed for confounding by registry and birth year. Signal detection cannot consider data quality, and we did not verify the timing of medication exposure. Several medications recommended for further investigation are also used to prevent premature labour/delivery (salbutamol, betamethasone, progesterone), which may be due to registration of medications prescribed after the first trimester. It was also not possible to assess the duration or dose of medications. A full literature review was not performed, and not all relevant literature may have been found. Further analyses of the EUROmediCAT dataset with verification of timing and dose are needed, along with analyses in similar datasets for further independent investigation into the signals identified within this analysis.

### 4.6 Conclusions

In this study, we identified 10 medication exposure–congenital anomaly signals (involving nine medications) warranting further investigation using the novel IC‐Bic‐BHM signal detection method. High‐ranking signals should be prioritized for investigation. These signals are hypothesis forming only and require further independent investigations to confirm any causal association.

## AUTHOR CONTRIBUTIONS

Conceptualization: Hannah Johnson, Helen Dolk, Maria Loane, Christine Damase‐Michel, Joanne Given, Hedvig Nordeng, Jorieke E. H. Bergman, Iain Carey and Joan Morris. Methodology: Hannah Johnson, Helen Dolk, Maria Loane, Christine Damase‐Michel, Joanne Given, Hedvig Nordeng, Jorieke E. H. Bergman, Iain Carey and Joan Morris. Software: Hannah Johnson and Joan Morris. Formal analysis: Hannah Johnson and Joan Morris. Data curation: Elly Den Hond, Ester Garne, Florence Rouget, Lea Bruneau, Isabelle Monier, Anke Rissmann, Mary O'Mahony, Miriam Gatt, Renée Lutke, Joanna Sichitiu, Elisa Ballardini, Alessio Coi, Clara Cavero Carbonell, David Tucker. Writing—original draft: Hannah Johnson, Iain Carey and Joan Morris. Writing—review and editing: Hannah Johnson, Helen Dolk, Maria Loane, Christine Damase‐Michel, Joanne Given, Hedvig Nordeng, Jorieke E. H. Bergman, Iain Carey, Elly Den Hond, Ester Garne, Florence Rouget, Lea Bruneau, Isabelle Monier, Anke Rissmann, Mary O'Mahony, Miriam Gatt, Renée Lutke, Joanna Sichitiu, Elisa Ballardini, Alessio Coi, Clara Cavero Carbonell, David Tucker and Joan Morris. Supervision: Helen Dolk, Maria Loane, Christine Damase‐Michel, Joanne Given, Hedvig Nordeng, Jorieke E. H. Bergman, Iain Carey and Joan Morris.

## CONFLICT OF INTEREST STATEMENT

The authors declare no conflicts of interest.

## Supporting information


**Figure S1:** Comparison of Results to Previous EUROmediCAT Signal Detection Studies.

**Table S1:** Included Medications.

**Table S2:** Included Anomalies.

**Table S3:** Top 20 Ranked Signals Generalised Linear Models.

**Table S4:** Full IC‐Bic‐BHM Results.

**Table S5.** Comparison of Previous EUROmediCAT Signal Detection Studies.

**Table S6:** Comparison of Signals to Previous EUROmediCAT Studies.

**Table S7:** Top 20 Ranked Signals Concurrent Exposures and Anomalies.

## Data Availability

EUROmediCAT encourages the use of its data and networks for pharmacovigilance and drug safety research following study approval from the Steering Group and member registries. Information on how to request data for a study is available at: https://euromedicat.eu/research/howtoproposeorcommissionspecificstudies. The data used to generate the results for this study are not publicly available due to potential disclosure.
