# Supplementary material for: Case‐malformed signal detection and prioritisation using EUROmediCAT data for pharmacovigilance in pregnancy
Source: Br J Clin Pharmacol. 2026 Jul 1;92(10):3586–97. doi: 10.1002/bcp.70645 (PMC13619031; doi:10.1002/bcp.70645)
Supplement: Supplementary file 1 [file BCP-92-3586-s002.docx]

| **Table S5. Comparison of Previous EUROmediCAT Signal Detection Studies** | | | |
| --- | --- | --- | --- |
| Characteristic | 1995-2011 Signal Detection | 1995-2015 Signal Detection | 2005-2018 Signal Detection |
| Number of Included Cases | 14,950 | 45,749 | 87,531 |
| Inclusion/Exclusion Criteria | - Excluding genetic conditions  - Excluding isolated hip dislocation  - Must be exposed to at least one medication (excluding folic acid, minerals, vitamins and/or topical medications coded to less than 5 digits) | - Excluding genetic conditions  - Excluding isolated hip dislocation  - Must be exposed to at least one medication (excluding folic acid, minerals, vitamins, topical medications and medications coded to less than 5 digits) | - Excluding genetic conditions  - Excluding isolated hip dislocation  - Excluding stillbirths and terminations from registries that cannot provide medication data for such cases |
| Method | Case-control approach Odds Ratio with 50% FDR and adjustment for registry | PRR with a double FDR process | IC-Bic-BHM with no adjustments |
| Exposures | 4^th^ and 5^th^ level ATC codes with at least 3 exposures | 4^th^ and 5^th^ level ATC codes with at least 3 exposures. | 5^th^ level ATC codes with at least 3 exposures excluding vitamins, minerals, and nutrients. |
| Outcomes | 59 anomaly subgroups | 61 anomaly subgroups | 61 anomaly subgroups |
| *Notes:*  *ATC: Anatomical-Therapeutic-Chemical medication coding. IC-Bic-BHM: Information component-Bicluster-Bayesian Hierarchical Modelling. Comparison of characteristics from current and previous EUROmediCAT studies.* | | | |
