# Supplementary material for: Case‐malformed signal detection and prioritisation using EUROmediCAT data for pharmacovigilance in pregnancy
Source: Br J Clin Pharmacol. 2026 Jul 1;92(10):3586–97. doi: 10.1002/bcp.70645 (PMC13619031; doi:10.1002/bcp.70645)
Supplement: Supplementary file 2 [file BCP-92-3586-s004.docx]

| **Table S7: Top 20 Ranked Signals Concurrent Exposures and Anomalies** | | | | | | | |
| --- | --- | --- | --- | --- | --- | --- | --- |
| Medication-Anomaly Pair | | Signal | | Ranking | | Annotation | |
| ATC Code /  Substance Name | AL Code /  Anomaly Name | Number Observed | IC-Bic-BHM (95% Credible Interval) | Estimated Additional Hospital Time per 100,000 Pregnancies (Days) | Rank | Concurrent Exposures | Concurrent Anomalies |
| A02BB01/  Misoprostol | AL4/  Anencephaly | 5 | 15 (5.2 – 32) | 1,959 | 1 | 4 cases. N02BE01 (3) | Isolated (4) |
| C09CA03/  Valsartan | AL53/  Bilateral renal agenesis | 4 | 22 (3.1 – 79) | 1,642 | 2 | 4 cases. No included medication with count ≥3. |  |
| R01AD01/  Belcometasone | AL53/  Bilateral renal agenesis | 3 | 1.2 (1.7 – 22) | 1,168 | 3 | ≥3 cases. No included medication with count ≥3. |  |
| H03AA01 /  Levothyroxine Sodium | AL41 /  Oesophageal atresia with or without fistula | 39 | 1.7 (1.2 – 2.3) | 1,047 | 4 | 11 cases. G03DA04 (4) | Isolated (18); AL21 (5); AL22 (4); AL44 (5); AL62 (3); AL112 (5) |
| H03AA01 /  Levothyroxine Sodium | AL24 /  Tetralogy of Fallot | 48 | 1.7 (1.3 – 2.3) | 829 | 5 | 13 cases. No included medication with count ≥3. | Isolated (37) |
| H03AA01 /  Levothyroxine Sodium | AL21 /  Ventricular septal defect | 452 | 1.1 (1.0 – 1.2) | 739 | 6 | 120 cases. A02BA02 (3); A10AB05 (4); A10AE04 (3); B01AB05 (5); B01AC06 (14); C02AB01 (3); C07AG01 (3); C08CA05 (5); C10AX06 (4); G03DA04 (15); G03DB01 (3); G03GA01 (5); H03CA (15); J01FA10 (4); J01XX01 (3); N02BA01 (6); N02BE01 (5); R03AC02 (3); R03BA01 (3); R03BA02 (4) | Isolated (317); AL8 (3); AL109 (3); AL19 (11); AL22 (48); AL23 (4); AL27 (12); AL28 (5); AL110 (11); AL30 (3); AL41 (5); AL44 (6);  AL55 (10); AL59 (8); AL62 (5); AL66 (4);  AL68 (4); AL79 (4); AL112 (5) |
| N02BE01 /  Paracetamol | AL50 /  Gastroschisis | 22 | 1.7 (1.1 – 2.3) | 700 | 7 | 5 cases. No included medication with count ≥3. | Isolated (16) |
| R03AC02 /  Salbutamol | AL50 /  Gastroschisis | 13 | 2.4 (1.3 – 4.0) | 578 | 8 | 8 cases. No included medication with count ≥3. | Isolated (11) |
| C08CA05 /  Nifedipine | AL41 /  Oesophageal atresia with or without fistula | 10 | 4.5 (2.3 – 8.0) | 507 | 9 | 7 cases. J01CA01 (3) | Isolated (3) |
| G03DB01 /  Dydrogesterone | AL22 /  Atrial septal defect | 36 | 2.8 (2.0 – 3.9) | 494 | 10 | 17 cases. A10BA02 (6); B01AC06 (9);  G03DA04 (3); R03AC02 (3) | Isolated (18); AL21 (10); AL27 (4) |
| G03DA04 /  Progesterone | AL22 /  Atrial septal defect | 74 | 1.4 (1.1 – 1.7) | 425 | 11 | 47 cases. A10BA02 (4); B01AB05 (3);  B01AC06 (7); G03CA03 (5); G03DB01 (3); H03AA01 (5); H03CA (3) | Isolated (34); AL21 (26); AL27 (5); AL32 (3) |
| H02AB01 /  Betamethasone | AL22 /  Atrial septal defect | 34 | 1.8 (1.3 – 2.6) | 422 | 12 | 13 cases. B01AB05 (3); C08CA05 (3);  G02CX01 (5); J01CA01 (3) | Isolated (17); AL21 (6) |
| N03AG01 /  Valproic acid | AL6 /  Spina bifida | 14 | 5.8 (3.3 – 9.5) | 349 | 13 | 8 cases. N03AX09 (5) | Isolated (7); AL66 (5) |
| A10AB05 /  Insulin aspart | AL19 /  Transposition of great vessels | 10 | 3.5 (1.7 – 6.2) | 346 | 14 | 9 cases. A10AC01 (4); A10AE04 (3) | Isolated (6); AL21 (3) |
| N03AX09 /  Lamotrigine | AL43 /  Atresia or stenosis of other parts of small intestine | 4 | 5.1 (1.5 – 12) | 344 | 15 | < 3 cases. No included medication with count ≥3. | Isolated (<3) |
| J01CA01 /  Ampicillin | AL41 /  Oesophageal atresia with or without fistula | 7 | 3.7 (1.6 – 7.2) | 335 | 16 | 6 cases. C08CA05 (3) | Isolated (3) |
| B01AC06 /  Acetylsalicylic acid | AL22 /  Atrial septal defect | 40 | 1.6 (1.1 – 2.1) | 318 | 17 | 29 cases. A10BA02 (3); B01AB05 (5);  G03DA04 (7); G03DB01 (9); H03AA01 (4) | Isolated (20); AL21 (9); AL27 (5) |
| N03AG01 /  Valproic acid | AL22 /  Atrial septal defect | 24 | 2.5 (1.7 – 3.7) | 309 | 18 | 15 cases. N03AX09 (3) | Isolated (8); AL21 (6); AL27 (5) |
| H03CA /  Iodine therapy | AL8 /  Severe microcephaly | 24 | 3.2 (2.1 – 4.7) | 296 | 19 | 12 cases. A02BA02 (3); C10AX06 (5);  G03DA04 (8) | Isolated (13); AL75 (3) |
| H03CA /  Iodine therapy | AL55 /  Congenital hydronephrosis | 56 | 1.7 (1.3 – 2.2) | 290 | 20 | 28 cases. B01AC06 (6); C10AX06 (8);  G03DA04 (7); H03AA01 (5); N02BE01 (6) | Isolated (49) |
| *Notes; ATC: Anatomical-Therapeutic-Chemical medication coding. IC-Bic-BHM: Information component-Bicluster-Bayesian Hierarchical Modelling. List of concurrent exposures and anomalies within observed cases for each signal.* | | | | | | | |
