# Supplementary material for: Case‐malformed signal detection and prioritisation using EUROmediCAT data for pharmacovigilance in pregnancy
Source: Br J Clin Pharmacol. 2026 Jul 1;92(10):3586–97. doi: 10.1002/bcp.70645 (PMC13619031; doi:10.1002/bcp.70645)
Supplement: Supplementary file 3 [file BCP-92-3586-s006.docx]

**Figure S1: Comparison of Results to Previous EUROmediCAT Signal Detection Studies**

*Notes: IC-Bic-BHM: Information Component-Bicluster-Bayesian Hierarchical Modelling. ATC: Anatomical-Therapeutic-Chemical medication coding. Comparison of signals identified within EUROmediCAT data by the IC-Bic-BHM with data from birth years 2005-2018, to two previous studies.*
