# Supplementary material for: Case‐malformed signal detection and prioritisation using EUROmediCAT data for pharmacovigilance in pregnancy
Source: Br J Clin Pharmacol. 2026 Jul 1;92(10):3586–97. doi: 10.1002/bcp.70645 (PMC13619031; doi:10.1002/bcp.70645)
Supplement: Supplementary file 4 [file BCP-92-3586-s008.docx]

| **Table S3: Top 20 Ranked Signals Generalised Linear Models** | | | | | | | | | |
| --- | --- | --- | --- | --- | --- | --- | --- | --- | --- |
| Medication-Anomaly Pair | | Signal | | Ranking | | Generalised Linear Model | | | |
| ATC Code /  Substance Name | AL Code /  Anomaly Name | Number Observed | IC-Bic-BHM (95% Credible Interval) | Estimated Additional Hospital Time per 100,000 Pregnancies (Days) | Rank | Included Registries  (Number with medication-anomaly pair / number with medication) | Number of Cases Excluded | Number of Cases Included | Odds Ratio, Adjusted for Birth Year and Registry (95% Confidence Interval) |
| A02BB01/  Misoprostol | AL4/  Anencephaly | 5 | 15 (5.2 – 32) | 1,959 | 1 | Emilia Romagna: (< 3 / < 3)  Northern Netherlands: (<8 / 8)  Funen: (< 3 / < 3)  Saxony Anhalt: (< 3 / < 3)  Paris: (< 3 / 3)  Isle de la Reunion: (< 6 / 6)  Valencian Region: (< 3 / < 3)  Brittany: (< 3 / 3) | 32,956 | 54,557 | 15 (5.7 – 41) |
| C09CA03/  Valsartan | AL53/  Bilateral renal agenesis | 4 | 22 (3.1 – 79) | 1,642 | 2 | Emilia Romagna: (< 3/ < 3)  Saxony Anhalt: (< 7/ 7)  Malta: (< 3/ < 3)  Paris: (< 3 / < 3)  Isle de la Reunion: (< 4 / 4)  Valencia Region: (< 3/ < 3) | 47,271 | 40,242 | 76 (23 – 258) |
| R01AD01/  Beclometasone | AL53/  Bilateral renal agenesis | 3 | 12 (1.7 – 22) | 1,168 | 3 | Northern Netherlands: (< 38 / 38)  Tuscany: (< 3 / < 3)  Wales: (< 5 / 5)  Saxony Anhalt: (< 3 / < 3)  Paris: (< 3 / < 3) | 47,314 | 40,199 | 13 (3.9 – 43) |
| H03AA01 /  Levothyroxine Sodium | AL41 /  Oesophageal atresia with or without fistula | 39 | 1.7 (1.2 – 2.3) | 1,047 | 4 | Antwerp: (< 3 / 94)  Emilia Romagna: (6 / 274)  Northern Netherlands: (< 3 / 101)  Funen: (< 3 / 15)  Tuscany: (5 / 236)  Vaud: (< 3 / 83)  Cork & Kerry: (< 3 / 22)  Malta; (< 3 / 59)  Wales: (< 3 / 156)  Saxony Anhalt: (5 / 249)  Paris: (4 / 176)  Isle de la Reunion: (< 3 / 32)  Valencian Region: (14 / 500)  Brittany: (< 3 / 226) | 0 | 87,513 | 1.7 (1.2 - 2.3) |
| H03AA01 /  Levothyroxine Sodium | AL24 /  Tetralogy of Fallot | 48 | 1.7 (1.3 – 2.3) | 829 | 5 | Antwerp: (< 3 / 94)  Emilia Romagna: (5 / 274)  Northern Netherlands: (< 3 / 101)  Funen: (< 3 / 15)  Tuscany: (6 / 236)  Vaud: (< 3 / 83)  Cork & Kerry: (< 3 / 22)  Malta; (< 3 / 59)  Wales: (7 / 156)  Saxony Anhalt: (3 / 249)  Paris: (6 / 176)  Isle de la Reunion: (< 3 / 32)  Valencian Region: (9 / 500)  Brittany: (6 / 226) | 0 | 87,513 | 1.8 (1.3 - 2.4) |
| H03AA01 /  Levothyroxine Sodium | AL21 /  Ventricular septal defect | 452 | 1.1 (1.0 – 1.2) | 739 | 3 | Antwerp: (11 / 94)  Emilia Romagna: (62 / 274)  Northern Netherlands: (14 / 101)  Funen: (< 3 / 15)  Tuscany: (60 / 236)  Vaud: (17 / 83)  Cork & Kerry: (< 3 / 22)  Malta; (7 / 59)  Wales: (22 / 156)  Saxony Anhalt: (38 / 249)  Paris: (27 / 176)  Isle de la Reunion: (6 / 32)  Valencian Region: (135 / 500)  Brittany: (48 / 226) | 0 | 87,513 | 1.1 (1.0 - 1.2) |
| N02BE01 /  Paracetamol | AL50 /  Gastroschisis | 22 | 1.7 (1.1 – 2.3) | 700 | 4 | Antwerp: (< 3 / 20)  Emilia Romagna: (< 3 / 113)  Northern Netherlands: (6 / 680)  Funen: (< 3 / 39)  Tuscany: (< 3 / 59)  Vaud: (< 3 / 44)  Cork & Kerry: (< 3 / 6)  Malta; (< 3 / 56)  Wales: (10 / 182)  Saxony Anhalt: (< 3 / 39)  Paris: (< 3 / 20)  Isle de la Reunion: (< 3 / 3)  Valencian Region: (< 3 / 105)  Brittany: (< 3 / 107) | 0 | 87,513 | 1.9 (1.2 - 3.0) |
| R03AC02 /  Salbutamol | AL50 /  Gastroschisis | 13 | 2.4 (1.3 – 4.0) | 578 | 5 | Antwerp: (< 3 / 8)  Emilia Romagna: (< 3 / 18)  Northern Netherlands: (< 3 / 103)  Funen: (< 3 / 13)  Tuscany: (< 3 / 12)  Vaud: (< 3 / 16)  Cork & Kerry: (< 3 / 64)  Malta; (< 3 / 51)  Wales: (9 / 283)  Saxony Anhalt: (< 3 / 6)  Isle de la Reunion: (< 3 / 7)  Valencian Region: (< 3 / 27) | 16,510 | 71,003 | 1.8 (1.0 - 3.2) |

| C08CA05 /  Nifedipine | AL41 /  Oesophageal atresia with or without fistula | 10 | 4.5 (2.3 – 8.0) | 507 | 6 | Antwerp: (< 3 / 3)  Emilia Romagna: (< 3 / 36)  Northern Netherlands: (< 3 / < 3) Tuscany: (< 3 / 16)  Vaud: (< 3 / 13)  Cork and Kerry: (< 3 / < 3)  Malta: (< 3 / < 3)  Wales: (< 3 / 13)  Saxony Anhalt: (< 3 / 5)  Paris: (< 3 / 3)  Isle de la Reunion: (< 3 / < 3)  Valencian Region: (8 / 83)  Brittany: (< 3 / 27) | 1,552 | 85,961 | 4.4 (2.3 - 8.3) |
| --- | --- | --- | --- | --- | --- | --- | --- | --- | --- |
| G03DB01 /  Dydrogesterone | AL22 /  Atrial septal defect | 36 | 2.8 (2.0 – 3.9) | 494 | 7 | Emilia Romagna: (< 3 / < 3)  Northern Netherlands: (< 3 / 3)  Tuscany: (< 3 / < 3)  Cork and Kerry: (< 3 / 3)  Malta: (35 / 165)  Paris: (< 3 / 5)  Brittany: (< 3 / < 3) | 44,523 | 41,438 | 1.1 (0.7 - 1.6) |
| G03DA04 /  Progesterone | AL22 /  Atrial septal defect | 74 | 1.4 (1.1 – 1.7) | 425 | 8 | Antwerp: (5 / 38)  Emilia Romagna: (21 / 229)  Northern Netherlands: (< 3 / 65) Tuscany: (< 3 / 79)  Vaud: (4 / 32)  Cork and Kerry: (< 3 / 25)  Malta: (19 / 62)  Wales: (< 3 / 33)  Saxony Anhalt: (6 / 34)  Paris: (< 3 / 29)  Valencian Region: (6 / 137)  Brittany: (< 3 / 12) | 5,031 | 82,482 | 1.2 (0.9 - 1.5) |
| H02AB01 /  Betamethasone | AL22 /  Atrial septal defect | 34 | 1.8 (1.3 – 2.6) | 422 | 9 | Antwerp: (< 3 / 3)  Emilia Romagna: (< 3 / 35)  Northern Netherlands: (< 3 / < 3)  Tuscany: (6 / 40)  Wales: (< 3 / 4)  Saxony Anhalt: (16 / 44)  Paris: (< 3 / < 3)  Isle de la Reunion: (< 3 / 3)  Valencian Region: (3 / 31)  Brittany: (8 / 39) | 9,067 | 78,446 | 2.0 (1.3 - 2.9) |
| N03AG01 /  Valproic acid | AL6 /  Spina bifida | 14 | 5.8 (3.3 – 9.5) | 349 | 10 | Antwerp: (< 3 / 9)  Emilia Romagna: (< 3 / 8)  Northern Netherlands: 4 / 13)  Funen: (< 3 / 6)  Tuscany: (< 3 / 4)  Vaud: (< 3 / 3)  Cork & Kerry: (< 3 / < 3)  Malta; (< 3 / 3)  Wales: (3 / 18)  Saxony Anhalt: (< 3 / 15)  Paris: (< 3 / 15)  Isle de la Reunion: (< 3 / 17)  Valencian Region: (< 3 / 11)  Brittany: (3 / 12) | 0 | 87,513 | 5.7 (3.3 - 10) |
| A10AB05 /  Insulin aspart | AL19 /  Transposition of great vessels | 10 | 3.5 (1.7 – 6.2) | 346 | 11 | Antwerp: (< 3 / 18)  Emilia Romagna: (< 3 / 7)  Northern Netherlands: (< 3 / 25)  Funen: (< 3 / 6)  Tuscany: (< 3 / < 3)  Vaud: (< 3 / 15)  Cork & Kerry: (< 3 / 7)  Malta; (< 3 / < 3)  Wales: (5 / 47)  Saxony Anhalt: (< 3 / 5)  Paris: (< 3 / 23)  Isle de la Reunion: (< 3 / 18)  Valencian Region: (< 3 / 10)  Brittany: (< 3 / 20) | 0 | 87,513 | 3.9 (2.1 - 7.2) |
| N03AX09 /  Lamotrigine | AL43 /  Atresia or stenosis of other parts of small intestine | 4 | 5.1 (1.5 – 12) | 344 | 12 | Antwerp: (< 3 / 5)  Emilia Romagna: (< 3 / < 3)  Northern Netherlands: (< 3 / 6)  Funen: (< 3 / 8)  Tuscany: (< 3 / 5)  Vaud: (< 3 / 4)  Cork and Kerry: (< 3 / 8)  Wales: (< 3 / 34)  Saxony Anhalt: (< 3 / 14)  Paris: (< 3 / 14)  Isle de la Reunion: (< 3 / 10)  Valencian Region: (< 3 / 11)  Brittany: (< 3 / 29) | 1,559 | 85,954 | 6.4 (2.3 - 18) |

| J01CA01 /  Ampicillin | AL41 /  Oesophageal atresia with or without fistula | 7 | 3.7 (1.6 – 7.2) | 335 | 13 | Emilia Romagna: (< 3 / 22)  Northern Netherlands: (< 3 / < 3)  Funen: (< 3 / < 3)  Tuscany: (< 3 / 13)  Saxony Anhalt: (< 3 / 20)  Isle de la Reunion: (< 3 / < 3)  Valencian Region: (6 / 113) | 42,796 | 44,717 | 3.3 (1.5 - 7.0) |
| --- | --- | --- | --- | --- | --- | --- | --- | --- | --- |
| B01AC06 /  Acetylsalicylic acid | AL22 /  Atrial septal defect | 40 | 1.6 (1.1 – 2.1) | 318 | 14 | Antwerp: (< 3 / 20)  Emilia Romagna: (< 3 / 49)  Northern Netherlands: (< 3 / 14)  Tuscany: (< 3 / 29)  Vaud: (< 3 / 31)  Malta: (20 / 73)  Wales: (< 3 / 16)  Saxony Anhalt: (< 3 / 1)  Paris: (< 3 / 10)  Isle de la Reunion: (< 3 / 3)  Valencian Region: (11 / 104)  Brittany: (< 3 / 10) | 4,453 | 83,060 | 1.2 (0.9 - 1.7) |
| N03AG01 /  Valproic acid | AL22 /  Atrial septal defect | 24 | 2.5 (1.7 – 3.7) | 309 | 15 | Antwerp: (< 3 / 9)  Emilia Romagna: (3 / 8)  Northern Netherlands: (3 / 13)  Funen: (< 3 / 6)  Tuscany: (< 3 / 4)  Vaud: (< 3 / 3)  Cork & Kerry: (< 3 / < 3)  Malta; (< 3 / 3)  Wales: (< 3 / 18)  Saxony Anhalt: (4 / 15)  Paris: (< 3 / 15)  Isle de la Reunion: (< 3 / 17)  Valencian Region: (3 / 11)  Brittany: (3 / 12) | 0 | 87,513 | 3.1 (2.0 - 4.7) |
| H03CA /  Iodine therapy | AL8 /  Severe microcephaly | 24 | 3.2 (2.1 – 4.7) | 296 | 16 | Saxony Anhalt: (3 / 98)  Valencian Region: (21 / 447) | 71,399 | 16,114 | 2.0 (1.3 - 3.1) |
| H03CA /  Iodine therapy | AL55 /  Congenital hydronephrosis | 56 | 1.7 (1.3 – 2.2) | 290 | 17 | Saxony Anhalt: (< 3 / 98)  Valencian Region: (54 / 447) | 71,399 | 16,114 | 0.7 (0.5 - 0.9) |
| N02BE01 /  Paracetamol | AL55 /  Congenital hydronephrosis | 109 | 1.3 (1.0 – 1.5) | 274 | 18 | Antwerp: (< 3 / 20)  Emilia Romagna: (15 / 113)  Northern Netherlands: (42 / 680)  Funen: (3 / 39)  Tuscany: (< 3 / 59)  Vaud: (6 / 44)  Cork & Kerry: (< 3 / 6)  Malta; (4 / 56)  Wales: (17 / 182)  Saxony Anhalt: (< 3 / 39)  Paris: (< 3 / 20)  Isle de la Reunion: (< 3 / 3)  Valencian Region: (13 / 105)  Brittany: (3 / 107) | 0 | 87,513 | 1.2 (1.0 - 1.5) |
| N02BE01 /  Paracetamol | AL44 /  Ano-rectal atresia and stenosis | 33 | 1.6 (1.1 – 2.2) | 271 | 18 | Antwerp: (< 3 / 20)  Emilia Romagna: (< 3 / 113)  Northern Netherlands: (21 / 680)  Funen: (< 3 / 39)  Tuscany: (< 3 / 59)  Vaud: (< 3 / 44)  Cork & Kerry: (< 3 / 6)  Malta; (< 3 / 56)  Wales: (5 / 182)  Saxony Anhalt: (< 3 / 39)  Paris: (< 3 / 20)  Isle de la Reunion: (< 3 / 3)  Valencian Region: (< 3 / 105)  Brittany: (< 3 / 107) | 0 | 87,513 | 1.6 (1.1 - 2.3) |
| G03DA04 /  Progesterone | AL51 /  Omphalocele | 12 | 2.0 (1.1 – 3.4) | 255 | 20 | Antwerp: (< 3 / 38)  Emilia Romagna: (< 3 / 229)  Northern Netherlands: (< 3 / 65) Tuscany: (3 / 79)  Vaud: (< 3 / 32)  Cork and Kerry: (< 3 / 25)  Malta: (< 3 / 62)  Wales: (< 3 / 33)  Saxony Anhalt: (< 3 / 34)  Paris: (< 3 / 29)  Valencian Region: (< 3 / 137)  Brittany: (< 3 / 12) | 5,031 | 82,482 | 2.7 (1.5 - 4.8) |
| *Notes: ATC: Anatomical-Therapeutic-Chemical medication coding. IC-Bic-BHM: Information component-Bicluster-Bayesian Hierarchical Modelling. Estimated additional cases, average time in hospital in 1st year of life; and estimated additional hospital time per 100,000 pregnancies rounded after calculation. Lethal anomalies (anencephaly and bilateral renal agenesis) given 365 days in hospital. For congenital constriction bands, the average number of days for all anomalies including chromosomal and genetic was used (17.4 days).* | | | | | | | | | |
